# Organizational Work-Home Culture and its Relations with the Work–Family Interface and Employees’ Subjective Well-being

**DOI:** 10.1007/s11482-022-10048-w

**Published:** 2022-03-19

**Authors:** Andrea Bobbio, Luigina Canova, Anna Maria Manganelli

**Affiliations:** Department of Philosophy, Sociology, Education and Applied Psychology, University of Padova, Via Venezia, 14, 35131 Padova, PD Italy

**Keywords:** Work-home culture, Work-home interface, Subjective well-being, Cross-sectional study, Mediation analysis

## Abstract

The two studies reported in this paper aimed to present and discuss both the validation of the Work-Home Culture (WHC) scale (Dikkers et al., *Work & Stress*, *21*(2), 155–172, 2007) in the Italian context (Study 1), and a relational model that links the WHC to subjective well-being via the mediation of three facets of the work-home interface: work-family conflict, work-family enrichment and work-family balance (Study 2). Heterogeneous samples of workers from different organisations took part in the cross-sectional studies. Substantial support was provided for the robustness of the factorial structure of the 18-item WHC scale with five factors (three support dimensions and two hindrance dimensions). Individuals’ perceptions of a supportive WHC that characterises the organisation they work for – particularly with respect to work-family issues and the use of family-friendly benefits – turned out to be positively associated with work-family enrichment and balance. Only organisational time demands, which is a hindrance dimension, was associated with work-family conflict. Moreover, our findings suggest that WHC is significantly associated with subjective well-being and that this association is largely indirect – through the facets of work-family interface – rather than direct. The results of the two studies represent a relevant achievement from the perspective of conducting future research using this measure in different socio-cultural environments and ad hoc interventions in the fields of organisational psychology and occupational health.

## Introduction

In the recent decades, Western countries have experienced several significant demographic, cultural and social changes that affected the relation between the domains of work and family. Particularly, the Italian society – which constitutes the background of the research described in this paper – has been characterised by an increase in women’s participation in the labour market (ISTAT, 2020a) and in the number of dual–income and single-parent families. All this, along with an increase in life expectancy, has led to a growing number of families that experience the concurrent demand of childcare and eldercare responsibilities (ISTAT, 2020b, c). These changes have highlighted the difficulty related to fulfilling work, family and household responsibilities simultaneously. In Italy, the externalisation of care is limited, and the public investment in care services intended to create a balance between work and family is scarce. Despite some recent updates in family policies (e.g., the possibility of parental leave for men and the expanded use of flexible working hours), the family is expected to handle the welfare of relatives (Naldini & Saraceno, 2011), similarly to other countries of Mediterranean Europe (Beham et al., 2014; Kovacheva et al., 2011).

A large portion of the literature revealed the negative effects of the interference between work and family on individual and organisational outcomes, such as reduced job and life satisfaction, augmented physical and psychological strain (De Simone et al., 2014), and turnover intentions (Frone, 2003). Other studies demonstrated that workplace social support (especially organisation- and supervisor-specific work-family support) had positive effects on the relationship between work and family (Kossek et al., 2011) and on individual and organisational outcomes, such as job satisfaction, organisational commitment, and negative effects on turnover intentions (Allen, 2001; Lyness et al., 1999; Thompson et al., 1999).

**Fig. 1:**
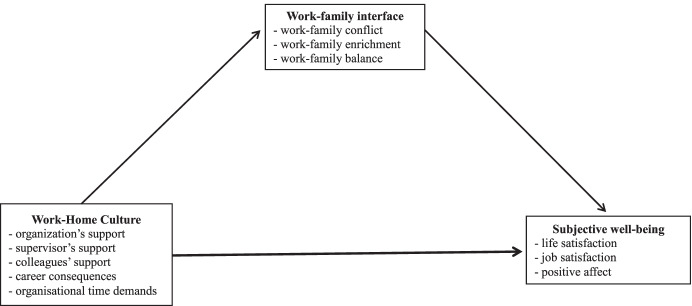
The hypothesized model of relations for Study 2

Workplace social support has also been conceptualised as a definite form of organisational culture: for example, family-friendly culture or work-family culture. These concepts refer to the degree to which an organisation’s culture supports its employees’ efforts to simultaneously manage work and personal commitments. Despite the acknowledgement of the importance of workplace culture, there is still a need to examine how the perceived workplace culture can contribute to employees’ well-being. With the intent of offering a contribution to the ongoing scientific debate on these relevant issues by providing results from the Italian context, we present two studies in this paper. The first one aimed to validate the 18-item Work-Home Culture (WHC) scale proposed by Dikkers et al. (2007); in the second study, we tested some models in which the three aspects of the work-family interface (i.e., conflict, enrichment and balance) mediated the relationships between WHC and employees’ subjective well-being (Fig. 1). Subjective well-being, which encompasses diverse aspects of people’s evaluation of their lives, is one of the constructs that have been proposed to operationalise the concept of workers’ well-being. Considering the changes currently occurring in the conditions and nature of work, workers’ well-being should become a priority for many organisations. A significant number of studies, in fact, proved that workers’ well-being might be a predictor of organisational ethics, performance, absenteeism and voluntary turnover (Wijngaards et al., 2021).

We argue that the studies presented in this paper can make several contributions to the current literature. First, a valid and reliable measure of the WHC for different cultural contexts is needed. Different countries can be characterized by different welfare systems with varying levels of statutory policies. Moreover, different contexts may influence both the type and the degree of work-family support and resources provided by the organizations, and thus shape workers’ perception of the organizational culture (Beham et al., 2014). The availability of a valid measure of WHC for different cultural contexts can be important for both scholars and practitioners – for instance, in the fields of social, work and organisational psychology and health and occupational medicine – to assess the construct with confidence. Further, it can also be important for using the scores generated from the administration of the scale in organisational projects that aim to implement policies and strategies that can help employees balance demands from multiple domains. Second, while a considerable amount of empirical evidence regarding the consequences of work-family conflict and enrichment exists, inquiries into the consequences of work-family balance are still scarce, as the research on the effects of the organisational WHC on the subjective well-being of employees.

## Study 1. Theoretical Background and Hypotheses

### Work-Home Culture

In response to the changes in the composition of the workforce, many organisations have implemented programs or policies through which employees can better balance demands from multiple domains. These programs, usually referred to as “family-friendly benefits”, include several options: for example, flextime, compressed workweek, job sharing, part-time work, parental leave, childcare facilities and smart working. However, sometimes, the implementation of these initiatives does not have the intended effect, especially when employees do not recognize a modification in the organisational norms and values, which usually discourages them from using these benefits (Allen, 2001). In fact, employees may fear the potential negative consequences associated with the advantages offered by the family-friendly benefits, such as negative judgments regarding their commitment to the organisation or worries that the use of these benefits will eventually jeopardise their career. Thus, formal policies and programs designed to help employees balance their work and family responsibilities may not achieve their purpose if the organisational culture does not legitimate and encourage their effective uptake (Thompson et al., 1999).

In the literature, different definitions and operationalisations of the concept of family-friendly culture can be found. However, one of the most used is that of Thompson et al. (1999), who stated that work-family culture is “the shared assumptions, beliefs, and values regarding the extent to which an organization supports and values the integration of employee’s work and family lives” (p. 394). Thompson et al. (1999) distinguished three components of work-family culture: *managerial support* (i.e., the extent to which managers are supportive and show sensitivity towards employees’ family responsibilities); *negative career consequences* (i.e., the perception that the use of family-friendly benefits may put future wage increase or promotions at risk), and *organisational time demands* (i.e., the expectation that employees should prioritise their work over their family and spend more time visibly at work). Thompson et al. (1999) also developed a 20-item scale that measured these three components of work-family culture. An organisational culture that is supportive of work–family issues can influence several work-related outcomes. Positive perceptions regarding work-family support increased organisational commitment (Lyness et al., 1999), decreased turnover intentions (Thompson et al., 1999), increased organisational citizenship behaviours (Bragger et al., 2005) and, finally, were positively associated with the use of family-friendly benefits (Thompson et al., 1999). Lo Presti et al. (2017), in a study that aimed to validate the scale proposed by Thompson et al. (1999) in the Italian context, found that a supportive work-family culture was positively correlated to job and family satisfaction.

Allen (2001) argued that it is important to disentangle the perceptions regarding organisational support from those regarding managerial support and proposed a fourth dimension: *supervisor’s support.* He defined a family-supportive supervisor as one who “is sympathetic to the employee’s desire to seek balance between work and family and who engages in efforts to help the employee accommodate his or her work and family responsibilities” (p. 417). Dikkers et al. (2004) argued also that support from colleagues should be considered and proposed a fifth component, called *colleagues’ support*, with regard to the use of work-family arrangements. Consequently, they introduced the concept of WHC, conceived as a five-dimensional construct, which included *organisation’s support*, *supervisor’s support*, *colleagues’ support*, *career consequences* and *organisational time demands*, and developed an 18-item scale to represent them. Of the 18 items, nine were adapted from the questionnaire developed by Thompson et al. (1999), while the other nine were newly developed.

Dikkers et al. (2007), in a study that was conducted in one public organisation and two private organisations in the Netherlands, hypothesised that these five components could be grouped into two higher-order dimensions: *support* and *hindrance*. According to Dikkers et al., (2004, p. 327): “Support refers to the extent to which the organization, direct supervisor, and colleagues are perceived to be supportive of the integration of employees’ work and private lives and the utilization of work-family arrangements. Hindrance reflects the extent to which organizational norms and expectations (i.e., time expectations and related negative career consequences) are perceived to impede employees’ work-home balance and the use of work-family arrangements. These two dimensions are expected to be negatively associated.” The results of the study by Dikkers et al. (2007) showed that the model with two second-order factors fitted the data well and that the structure of the WHC scale was invariant across organisations, genders and parental status. As expected, the two WHC dimensions were negatively related, and workers from public organisations reported experiencing higher levels of *support* and lower levels of *hindrance* than those from private organisations. Further, men and women, workers with and without children did not differ in their perceptions regarding WHC. Dikkers et al. (2007) examined the associations between the two second-order dimensions and the use of four specific arrangements (i.e., flextime, working part-time, subsidised childcare and parental leave): the results indicated that workers who reported experiencing higher levels of *support* were more likely to use the flextime, part-time work and subsidised childcare facilities. The WHC was not significantly related to the use of parental leave, which was most strongly predicted by gender (Dikkers et al., 2007).

To our knowledge, although some subscales or items of the WHC scale have been used in studies conducted in the Netherlands (Pas et al., 2011; Straub et al., 2018) or in other European countries (Beham et al., 2011, 2014), and in Australia (Timms et al., 2015), no one has further validated the factorial structure of the whole 18-item WHC scale. Some of these studies deepened the relations between specific dimensions of WHC, organizational variables or selected aspects of the work-home interface. As regards the organizational outcomes, Timms et al. (2015) highlighted the importance of a supportive culture (*organization’s* and *supervisor’s support*) for attenuating employees’ turnover intentions and psychological strain, and for increasing work engagement. Instead, *organizational time demands* and *negative career consequences* were predictive of turnover intentions; *negative career consequences* predicted also psychological strain. In line with these findings, Straub et al. (2018) found that perceptions of a supportive WHC strengthened employees work engagement and diminished their turnover intentions. In contrast, a hindrance organization culture resulted in lower employees’ work engagement and higher turnover intentions.

Despite the interest towards organisational strategies that enable employees to better balance demands from work and family domains, and the perceptions of employees regarding the implications of using such strategies, the WHC is a relatively under-researched concept.

Study 1 of this paper aimed to test the factorial structure of the Italian version of the WHC scale and evaluate the goodness-of-fit of a series of alternative factor models, following the original work of Dikkers et al. (2007). Further, in line with the steps followed by Dikkers et al. (2007), the robustness of the factorial structure of the WHC scale and the invariance of the parameter estimates (i.e., factor loadings, factor covariance and item error variances) were tested across samples representing differences in gender, parental status and public *vs* private sector organisations. We put forward the following hypotheses:

- H1: The WHC will be captured by five first-order dimensions (*organisation’s support*, *supervisor’s support*, *colleagues’ support*, *career consequences* and *organisational time demands*) and two second-order dimensions (*support* and *hindrance*).
- H2: The factorial structure will be invariant across all the sub-samples considered in the study.

Since previous research has shown that public organisations were more concerned with assisting their workers with care-giving responsibilities than companies in the private sector (Dikkers et al., 2007; Mauno et al., 2005), we expected the following:

- H3: Workers from public organisations will report higher levels of supportive WHC and lower levels of hindrance than those from private organisations.
- H4: In line with the results of Dikkers et al. (2007), we did not expect gender-based and parental status-based differences in WHC perceptions.

## Study 2. Theoretical Background and Hypotheses

Study 2 aimed to analyse the direct and indirect relationships between the five dimensions of WHC and subjective well-being via the mediation of the work-family interface (Fig. 1). Three different aspects of the work-family interface were considered: work-family conflict, work-family enrichment and work-family balance. Empirical studies examining the antecedents of work-family interface and work-related outcomes mainly draw upon the job demands-resources model (Demerouti et al., 2001), according to which job demands refer to “physical, social, or organizational aspects of a job that require sustained physical or mental effort and are therefore associated with certain physiological and psychological costs” (Demerouti et al., 2001, p. 501). Job resources refer to the physical, psychological, social and organisational aspects that serve to achieve work goals, stimulate personal growth and reduce the costs associated with job demands. Resources can be classified as external (social and organisational) and internal (cognitive features and action patterns) (Demerouti et al., 2001). Past research has identified certain job demands and external social resources that deplete or enhance the ability of employees to reconcile the work and family domains (e.g., Beham et al., 2011). For example, time-based demands (e.g., long working hours and overtime) are related to higher levels of work-home interference and lower satisfaction with work-family balance (Beham & Drobnič, 2010; Beham et al., 2011, 2014; Valcour, 2007). At the same time, social resources (e.g., a supportive work-family culture, a supportive supervisor, supportive co-workers and work-family policies) were found to be negatively related to work-home interference and positively related to satisfaction with work-family balance (Beham & Drobnič, 2010; Beham et al., 2011, 2014; Dikkers et al., 2007; Thompson et al., 1999; Valcour, 2007).

### Work-Family Interface: Conflict, Enrichment and Balance

The expression “work-family interface” describes a research field dealing with the relationships between the domains of work and family, which can be both positive and negative. Nevertheless, the conflict dominated work-family interface research for the last two decades of the twentieth century. This view originated from role theory (Merton, 1957) and from Goode’s (1960) role strain hypothesis. The work-family conflict was the core construct of the conflict perspective and it was defined as “a form of inter-role conflict in which the role pressure from work and family are mutually incompatible in some respect. That is, participation in the work (family) role is made more difficult by virtue of participation in the family (work) role” (Greenhaus & Beutell, 1985, p. 77).

The considerable amount of research on the effects of work-family conflict has revealed that this kind of conflict is negatively related to indicators of subjective well-being, such as job, family and life satisfaction (e.g., De Simone et al., 2014). Moreover, it is positively related to psychological strain, depression and burnout (e.g., Allen et al., 2000); it is also negatively associated with organisational commitment (e.g., Allen et al., 2000) and organisational citizenship behaviours (e.g., Bragger et al., 2005), and positively associated with turnover intentions (e.g., Allen et al., 2000; Frone, 2003).

With regard to the relationship between work-family culture and work-family conflict, Thompson et al. (1999) found that more supportive cultures were associated with less work-family conflict, and that less negative perceptions regarding the *career consequences* of using the benefits and lower *organisational time demands* were associated with less work-family conflict. Dikkers et al. (2007) analysed the relations between the two second-order dimensions of WHC and the four components of the work-home interaction – both negative (i.e., negative influence of work on home and vice versa) and positive (i.e., positive influence of work on home and vice versa) (Geurts et al., 2005). Employees who perceived higher levels of *support* for work-family issues experienced less interference from work on home, a more positive influence from work on home, and a more positive influence from home on work. The perceived *hindrance* was only linked to a negative component of the work-home interaction, which is the interference from work on home. Similar findings are reported by Beham et al. (2011) which found that *organizational time demands* were positively related to interference from work on home, while *supervisor’s* and *colleagues’ support* were negatively related to the same outcome.

With regard to the relationships between the dimensions of WHC and the work-family conflict, our hypotheses were as follows:

- H5: The three dimensions of work-home support (i.e., *organisation*, *supervisor* and *colleague* support) will be negatively associated with work-family conflict.
- H6: The two dimensions that characterise a hindering WHC (i.e., *negative career consequences* and *organisational time demands*) will be positively associated with work-family conflict.

More recently, scholars have focused their attention on the positive interactions between the roles of work and family. Several constructs were proposed, such as work-family enrichment, work-family positive spillover and work-family facilitation. Work-family enrichment is defined as “the extent to which experiences in one role improve the quality of life, namely performance or affect, in the other role” (Greenhaus & Powell, 2006, p. 73). Previous research has confirmed that work-family enrichment and work-family conflict are conceptually and empirically distinct, the correlation between them being found to be null or weak (e.g., Carlson et al., 2006; Ghislieri et al. 2011). With respect to the outcomes, work-family enrichment has been found to be positively related to an individual’s physical and mental health, job and family satisfaction, affective commitment (e.g., Baral & Bhargava, 2010; McNall et al., 2010; Wayne et al., 2006), and organisational citizenship behaviour (e.g., Bhargava & Baral, 2009). Regarding the relationships with the work-family culture, Wayne et al. (2006), using the scale by Thompson et al. (1999), found that *organisational time demands* were negatively related to work-family enrichment. Other studies highlighted that supervisors’ and co-workers’ support was linked to higher levels of work-family enrichment (e.g., Baral & Bhargava, 2010; Beham et al., 2011).

With respect to the relationships between the dimensions of WHC and the work-family enrichment, our hypotheses were as follows:

- H7: The three dimensions of work-home support will show positive associations with work-family enrichment.
- H8: The two dimensions of a hindering WHC will be negatively associated with work-family enrichment.

In the last decade, especially due to the emerging interest in the themes and practices that aim to reconcile work and family life, certain authors have focused their studies on the concept of work–family balance. Initially, work-family balance was equated to the absence of work-family conflict. Subsequently, Frone (2003) defined balance as the simultaneous experience of low conflict and high enrichment. More recently, other scholars have argued that balance is a concept that is distinct from both conflict and enrichment, and many different definitions have been proposed (for a review, see Sirgy & Lee, 2018; Wayne et al., 2017). Among them, some considered balance to be a global evaluation of the interplay between work and family (*global balance approaches*) (Wayne et al., 2017). Grzywacz and Carlson (2007) defined it as the “accomplishment of role-related expectations that are negotiated and shared between an individual and his/her role-related partners in the work and family domains” (p. 458). This view, wherein work-family balance is inextricably linked to the social context, does not necessarily imply that an individual should be a “superstar” in both work and family contexts but, rather, that he/she meets the basic expectations of both roles. According to Carlson et al. (2009), work-family balance is more global than individual experiences of conflict and enrichment, as it places emphasis on the individual’s ability to fulfil his/her responsibilities associated with both work and family domains and reflects his/her beliefs that significant others perceive him/her as being capable of accomplishing work and family responsibilities. Carlson et al. (2009) proposed a measure of work-family balance based on the theoretical definition of balance proposed by Grzywacz and Carlson (2007) and demonstrated that it was distinct from work-family conflict and enrichment. Recently, Landolfi and Lo Presti (2020) validated this measure in the Italian context, effectively supporting the distinction between work-family conflict, enrichment and balance. Their results also showed that work-family balance was negatively associated with work-family conflict and positively associated with work-family enrichment.

Although the concept of balance is extremely popular in the work-family literature, the empirical research focusing on work-family balance-related consequences is still limited when compared to that on the consequences of work-family conflict and enrichment. However, some evidence of the links between work-family balance and several positive work outcomes does exist. For instance, work-family balance was found to be positively associated with job and life satisfaction, organisational commitment and organisational citizenship behaviours and negatively associated with anxiety and depression (Carlson et al., 2009, 2013; Haar, 2013; Haar et al., 2014; Wayne et al., 2017). As regards the relations between WHC and work-family balance, very few evidence is available. For example, Beham and Drobnič (2010) found that high *organizational time demands* were negatively related to satisfaction with work-family balance (Valcour, 2007), while Beham et al. (2014) reported that *supervisor’s* and *colleagues’ support* were positively associated with the same aspect of work-home interface.

Our hypotheses concerning the relations between the dimensions of WHC and the work-family balance, were as follows:

- H9: The three supportive dimensions of WHC will show positive associations with work-family balance.
- H10: The two dimensions of a hindering WHC will be negatively associated with work-family balance.

### Subjective Well-Being, Work-Family Interface and Work-Home Culture

According to Diener (2000, 2013), subjective well-being is a broad construct that includes several correlated but distinct components, such as “life satisfaction (global judgment of one’s own life), satisfaction with important domains (e.g., work satisfaction), positive affect (experiencing of many pleasant emotions and moods), and low levels of negative affect (experiencing of unpleasant emotions and moods)” (Diener, 2000, p. 34). Life satisfaction is a trait-like, context-free construct; affect represents people’s evaluations of the events that occur in their lives and comprises both trait-like and state-like components (Wijngaards et al., 2021). Therefore, subjective well-being can be defined as an individual’s cognitive and affective appraisals of his/her life and includes experiencing pleasant emotions, a low level of negative moods and a high degree of life satisfaction.

Previous research that analysed the relationship between the work-family interface and subjective well-being found that work-family conflict was negatively associated with life satisfaction and affective components of subjective well-being (psychological strain, depression and anxiety), especially in the short term (Matthews et al., 2014); work-family enrichment was found to predict higher job satisfaction and overall life satisfaction (Ford et al., 2018).

As aforementioned, some empirical evidence supports the existence of a link between the work-family interface and subjective well-being, especially between work-family conflict and lower job and life satisfaction, negative affect or high psychological strain and between work-family enrichment and balance, higher satisfaction and lower psychological strain. Therefore, we postulated the following:

- H11: Work-family conflict will be negatively associated with subjective well-being.
- H12: Work-family enrichment and work-family balance will be positively associated with subjective well-being.

Other empirical evidence demonstrated that the work-family culture has an effect on work-related stress. For example, Thompson and Prottas (2005) showed that supportive supervisors and co-workers, and employees’ perception that they can use family-friendly benefits without fearing negative job or career consequences, were linked to lower levels of stress and depressive symptoms and greater positive work-family affective spillover.

Mauno et al. (2005), using three subscales adapted from the work-family scale by Thompson et al. (1999), found that an unsupportive work-family culture was associated directly and indirectly, through work-family conflict, to self-reported employees’ distress in two organizations (one public and one private). Beauregard (2011) discovered that none of the work-family culture dimensions had a significant direct relationship with strain when all the other dimensions were controlled, but work-home interference was found to fully mediate the effects of *organisational time demands* on strain for women and, partially, for men. Thus, the demand that requires employees to subjugate their personal lives to fulfil their work responsibilities was related to the amount of work-home interference they experienced, which, in turn, predicted increased levels of anxiety, fatigue and depression. Further, Ferguson et al. (2012) found that work-family balance partially mediated the relationship between co-workers’ support and job satisfaction.

Although organisational work-family culture has been demonstrated to be linked to work-related stress, little is known about the relationship between the five dimensions of WHC scale and employees’ subjective well-being. Additional studies are needed to gain better comprehension of the mechanism that can potentially explain how the WHC contributes to employees’ well-being. In particular, the key question is whether the WHC and subjective well-being are directly connected or whether other variables act as mediators of their relationship. In our research, we posit that the work-family interface is one mechanism through which organisations’ WHC exerts its effect on employees’ subjective well-being (Fig. 1). Hence, we hypothesised that the three facets of the work-home interface will mediate the relationships between the five dimensions of the WHC and subjective well-being (H13).

## Study 1

### Aims

Following Dikkers et al.’s (2007), Study 1 aims a) to evaluate the construct validity of the Italian version of the WHC scale; b) to test the invariance of the factorial structure across different subgroups of participants; c) to examine mean differences associated with the dimensions of the WHC scale across the same subgroups.

## Method

### Procedure and Participants

A heterogeneous sample of workers from different organisations took part in the study. Eight bachelor’s and master’s psychology students assisted with data collection as a part of their internship or research experience assignment. Using snowball sampling, each student recruited about 100–120 participants. The students were trained regarding the inclusion criterion (i.e., participants had to be employees who had a supervisor and, at least, one colleague) and regarding the ethical standards related to the recruitment process. The participants were informed about the aim of the study, the average duration of the task and the possibility of withholding their consent to participate in the research at any time and were assured that all their responses would remain confidential. Participants filled in the questionnaire individually and returned it on the same day that they had received it. The students returned the completed questionnaires – which were in sealed envelopes – to the researchers.

We collected 940 questionnaires, which were then carefully examined.^1^ We retained those that belonged to participants aged between 25 and 67, employees of either public or private companies and those who did not present missing data on the WHC scale items. Data collection was completed before the sanitary emergency caused by SARS-COV-2 (COVID 19) started.

The final sample comprised 784 participants: 309 men (39.4%), 472 women (60.2%) and three participants (0.4%) who did not indicate their gender. One hundred and twenty-eight participants (16.3%) were 25–29 years old, 404 (51.5%) were 30–49 years old, and 250 (31.9%) were over 50 years old. Two (0.3%) respondents did not indicate their age. Further, 13.5% participants had completed compulsory education (*n* = 106), 69.3% had an upper-secondary diploma (*n* = 544), and 17% had a college degree (*n* = 133); one participant did not indicate the level of education. Most participants worked in the private sector (*n* = 517, 65.9%), and 208 (26.5%) worked in public organisations; for 59 participants (7.5%), this information was not provided. Furthermore, 308 (39.3%) worked as clerical workers, 135 (17.2%) were blue-collar workers, 111 (14.2%) were health professionals, 79 (10.1%) were schoolteachers, 101 were employed in the service sector (12.9%), and 43 (5.5%) held a middle-management position (seven missing values). Three hundred and five respondents (38.9%) had been working for five years or less, 104 (13.3%) had a seniority between six and ten years, and 374 (47.7%) had been working for more than eleven years; one participant did not answer this question. More than a half of the participants (*n* = 521, 66.5%) had a permanent full-time job position; 132 (16.8%) had a permanent part-time position, and 128 (16.3%) had a fixed-term employment contract (three missing values). A majority of the participants (*n* = 468, 59.7%) reported having children (two missing values), and 61.7% were married or cohabiting (*n* = 484). The percentage of participants who lived in Northeast Italy was equal to 62.2% (*n* = 488), 14% lived in Southern Italy (*n* = 110), 11.6% lived in Northwest Italy (*n* = 91), and 10.7% (*n* = 84) lived in the Central Italy area (11 missing values).

### Measures

The translation process of the WHC scale included both the forward and the backward steps and the pilot test to gather feedback on the readability and content validity of the translated scale. The instrument was administered to 10 individuals, and no significant word changes were made. The Italian version is available in Appendix 1, Table A.

#### Work-Home Culture

It was measured with the 18-item instrument developed by Dikkers et al. (2007). The 18 items represented its five components: (a) *organisation’s support* (four items, e.g., “In this organisation, it is considered important that, beyond their work, employees have sufficient time left for their private life”); (b) *supervisor’s support* (four items, e.g., “My superior supports employees who want to switch to less demanding jobs for private reasons”); (c) *colleagues’ support* (four items, e.g., “My colleagues support employees who want to switch to less demanding jobs for private reasons”); (d) *career consequences* (four items, e.g., “To turn down a promotion for private reasons will harm one’s career progress in this organisation”); (e) *time demands* (three items, e.g., “To get ahead in this organisation, employees are expected to put their job before their private life when necessary”). The Likert-type answer alternatives ranged from 1 (completely disagree) to 7 (completely agree), with higher scores entailing higher levels of support, negative career consequences and time demands.

#### Socio-Demographics Variables

Gender, age, geographic area of residence, education, employment status and tenure were assessed.

#### Data Analysis

After verifying the univariate normality of items’ distributions using the skewness and kurtosis indices, we checked Mardia’s (1970) coefficient to assess the multivariate normality of the items’ distributions. To test the factorial structure of the WHC scale, we applied the confirmatory factor analysis (CFA), implemented via Lisrel 8.8. A maximum likelihood method of estimation was adopted based on the covariance matrix between the 18 items. To test the hypothesis that five first-order dimensions and two second-order dimensions (*support* and *hindrance*) characterise the WHC, we compared the fit of different factor models. Model fit was evaluated using chi-square, comparative fit index (CFI), root mean square error of approximation (RMSEA) and standardised root mean square residual (SRMR) indices according to the cut-off values that are accepted in the literature. Usually, a satisfactory model is denoted by nonsignificant χ^2^, CFI > 0.95, RMSEA ≤ 0.06 and SRMR ≤ 0.08 (Hu & Bentler, 1999). To compare non-nested models, we used the Akaike information criterion (AIC): lower AIC coefficients generally indicate better model fit (Schermelleh-Engel et al., 2003). To estimate reliability, composite reliabilities (CR) and Cronbach’s alpha coefficients were determined. Multi-group confirmatory factor analyses were performed for testing configural, metric, factor covariance and residual invariance across samples defined by gender, parental status and employment in a public *vs* private sector organisation. Since our sample contained more than 300 people, a change equal to or higher than -0.010 in CFI, supplemented by a change equal to or higher than 0.015 in RMSEA or a change equal to or higher than 0.030 in SRMR, would indicate non-invariance (Cheung and Rensvold 2002; Chen, 2007). Finally, the differences between the composite scores of subgroups were tested using multivariate analyses of variance (MANOVA) via SPSS 27.

## Results

### Confirmatory Factor Analysis

The skewness for all item distributions fell between -0.578 and -0.016, kurtosis fell between -1.191 and -0.437, and they did not highlight severe violations. Mardia’s coefficient was equal to 1.270. As it was not significant (< 1.96), it supported the multivariate normality of the items’ distributions.

Table 1 reports the fit indices for the models that were tested. Model 1, in which all the items of the WHC scale loaded on one factor, and Model 3, in which the five first-order factors loaded on one second-order factor, did not present adequate fit indices. Model 2a, with five first-order correlated factors, and Model 4a, in which the five first-order factors loaded on two second-order factors, reflecting *support and hindrance*, fit the data in an acceptable way. The CFI and SRMR fit indices of Model 2a and Model 4a were adequate, but the RMSEA was higher than the threshold of 0.06. To determine what could have caused the inflation of the RMSEA, we inspected the modification indices and found that the model could be improved by estimating the error covariance between three pairs of items, namely 1–2, 5–6 and 12–13 (see Table A in Appendix 1). Most probably, these were perceived to have some degree of overlap in meaning or wording or were placed close together in the questionnaire. The fit indices of the two models with correlated errors (M2b and M4b) were only slightly more adequate; in fact, a RMSEA value of 0.08 is considered acceptable (Schermelleh-Engel et al., 2003). Therefore, for parsimony we preferred to continue the analyses by considering the solution that did not have correlated errors.

**Table 1:** Factorial models of the WHC scale (*n* = 784)

Model	χ^2^	*df*	RMSEA	CFI	SRMR	AIC
M1 (one-factor model)	6010.41	135	0.24 90% CI: [0.23, 0.24]	0.70	0.16	6082.41
M2a (five-factor model)	1088.82	125	0.09 90% CI: [0.09, 0.10]	0.94	0.06	1180.82
M2b (five-factor model, correlated errors)	728.71	122	0.08 90% CI: [0.07, 0.08]	0.96	0.05	826.71
M3 (five first-order and one second-order factors model)	1275.42	130	0.11 90% CI: [0.10, 0.11]	0.92	0.11	1357.42
M4a (five first-order and two second-order factors model)	1106.46	129	0.09 90% CI: [0.09, 0.010]	0.94	0.07	1190.46
M4b (five first-order and two second-order factors model, correlated errors)	763.32	126	0.08 90% CI: [0.07, 0.08]	0.96	0.06	853.32

The standardised factor loadings (λ) for M2a were all statistically significant (*p* < 0.001) and ranged from 0.41 (item 18) to 0.93 (item 10) (see Table A in Appendix 1).

The average variance extracted (AVE) (Table 2), which reflects the overall amount of shared variance among the indicators that measure a latent construct, ranged from 0.51 to 0.69, exceeding the acceptable threshold level of 0.50 (Bagozzi & Yi, 1988). The correlations between the five latent factors ranged from small to large in size, and the square roots of the AVE were higher than the correlations between latent factors, thus indicating that the five factors were correlated but distinct (Fornell & Larcker, 1981). The composite reliability values of all latent constructs ranged from 0.80 to 0.89, which were above the acceptable level of 0.70 (Bagozzi & Yi, 1988). Further, the results of the internal reliability, calculated using Cronbach’s alpha values, were satisfactory.

**Table 2:** Descriptive statistics, reliability estimates and correlations between latent factors (*n* = 784)

Variables	M (*SD*)	Alpha	AVE	CR	1	2	3	4	5
1.Organisation’s support	4.17 (1.47)	0.89	0.68	0.89	-				
2.Colleagues’support	4.44 (1.33)	0.85	0.60	0.85	0.45	-			
3.Supervisor’s support	3.71 (1.47)	0.84	0.69	0.87	0.64	0.62	-		
4.Time demands	4.08 (1.64)	0.83	0.64	0.84	-0.28	-0.14	-0.22	-	
5.Career consequences	3.99 (1.35)	0.78	0.51	0.80	-0.31	-0.14	-0.27	0.64	-

AVE = Average variance extracted; CR = composite reliability. All correlation coefficients are significant with *p* < 0.01.

With regard to M4a, the correlation between the two second-order factors was equal to -0.36, and all gamma coefficients (γ) were significant, ranging from 0.91 (*supervisor’s support* on *support* dimension) and 0.67 (*colleagues’ support* on *support* dimension). The alpha coefficient for *support* was equal to 0.90, while that for *hindrance* was 0.83.

The comparison of the AIC indices of M2a and M4a revealed small differences, and this led us to prefer the model with the lowest AIC, M2a, which had five first-order correlated factors. Additionally, a WHC scale qualified by five scores can offer more details for both theoretical and applicative purposes.

In sum, the factor analyses supported the distinction between the five dimensions of the WHC scale. Moreover, given the small differences in data fit between M2a and M4a, the results of Dikkers et al. (2007) were corroborated and the use of the two comprehensive measures of *support* and *hindrance* was also legitimised.

### Measurement Invariance

Invariance was examined with reference to M2a (five correlated latent factors) and by contrasting the following subgroups of participants: male (*n* = 309) *vs* female (*n* = 472), parents (*n* = 468) *vs* nonparents (*n* = 314) and public-sector workers (*n* = 208) *vs* private-sector workers (*n* = 517). The multi-sample procedure was applied with four consequential hypotheses: i) *configural invariance* that represents the baseline model and requires an identical number of both the factors and the pattern of factor–item relations across the two groups; ii) *metric invariance* that requires all factor loading parameters (λ_y_) to be invariant across groups; iii) *invariance of construct covariance* that requires the relationships among constructs to be the same across groups; iv) *residual variance invariance* that indicates that scale items measure the latent constructs with the same degree of measurement error (θ_ε_). The configural and metric invariance were supported in all three multi-group comparisons: the χ^2^ difference tests were nonsignificant; all ΔCFIs were smaller than -0.01, and the differences between the RMSEA and SRMR values were below 0.015 and 0.030, respectively (Chen, 2007; Cheung & Rensvold, 2002). In the comparison based on gender, the factor covariance invariance and residual invariance were also supported. With regard to parental status, by constraining the factor covariance to be equivalent across both groups, the χ^2^ value increased and the fit decreased; however, the differences associated with the CFI, RMSEA and SRMR values were below the cut-off for rejecting invariance tests, and, therefore, the invariance could be considered as being supported. The invariance of the construct covariance cannot be seen as entirely supported in the comparison between public *vs* private organisations: in this case, Δχ^2^ is significant, and ΔCFI is equal to -0.01; additionally, the residual variance invariance is also not completely supported. Considering all the evidence and that “more advanced levels of invariance represent very strict standards that are often difficult to fulfil in practice” (Chen, 2007, p. 466), we concluded in favour of the overall measurement invariance of the Italian version of the five-correlated-factor WHC scale (for more details, please see Table B in Appendix 2).

### Work-Home Culture and Demographic and Organisational Characteristics

Using MANOVA, we examined the mean differences associated with the five dimensions of the WHC across the gender, parental status and private *vs* public sectors subgroups. As for gender, the main multivariate effect was significant (*F*_5,775_ = 4.11, *p* < 0.002, *η*^*2*^_*par*_ = 0.03). The univariate effects were significant for *time demands* (*F*_1,779_ = 9.30, *p* < 0.003, *η*^*2*^_*par*_ = 0.01) and *career consequences* (*F*_1,779_ = 7.77, *p* < 0.006, *η*^*2*^_*par*_ = 0.01). In both cases, men presented higher scores (4.30 and 4.15, respectively) than women (3.94 and 3.88, respectively). The main multivariate effect of parental status was not significant (*F*_5,776_ = 1.19, *ns*)*.* Workers from the public and private sectors had different perceptions regarding WHC, since the main multivariate effect was significant (*F*_5,719_ = 8.40, *p* < 0.0001, *η*^*2*^_*par*_ = 0.06)*.* The univariate effects were significant for *organisation’s support* (*F*_1,723_ = 13.63, *p* < 0.0001, *η*^*2*^_*par*_ = 0.02), *supervisor’s support* (*F*_1,723_ = 4.48, *p* < 0.04, *η*^*2*^_*par*_ = 0.01), *time demands* (*F*_1,723_ = 9.42, *p* < 0.003, *η*^*2*^_*par*_ = 0.01) and *career consequences* (*F*_1,723_ = 9.24, *p* < 0.003, *η*^*2*^_*par*_ = 0.01). In all cases, workers from private organisations presented higher mean scores than those from the public sector (4.26, 3.78, 4.20, 4.09 *vs* 3.81, 3.52, 3.78, 3.75, respectively).

### Conclusion

In line with H1, the results of the CFAs attested that the WHC scale designed by Dikkers et al. (2007) can be represented both by a five first-order-factor structure and by a structure made up of five first-order factors and two second-order factors. Additionally, the five-factor structure can be considered as invariant across gender, parental status and type of organisation (H2). Overall, these results underline the robustness of the scale in the Italian context.

Regarding organisation characteristics, our results partially supported H3, since they highlighted that employees from public organisations perceived the WHC as being less supportive and less obstructive with respect to establishing a balance between work and home duties as compared to those from private organisations. That is, employees of public organisations perceived less *time demands* and less negative *career consequences* in relation to their efforts to manage both work and family duties; but at same time, they feel less supported by both the organisation and their supervisor with regard to managing these efforts. Even H4 was only partially supported. The respondents did not differ in terms of their perception of the WHC of their organisations regardless of whether they had children; on the contrary, men (*vs* women) perceived their organisations as being more obstructive to establishing a balance between work and home duties in terms of both *time demands* and negative *career consequences*.

## Study 2

### Aims

Study 2 aims to test some hypotheses regarding the relationships between the five dimension of WHC, three aspects of the work-home interface and three features of subjective well-being (see Fig. 1).

## Method

### Procedure and Participants

Study 2 was also conducted using a heterogeneous sample of workers belonging to different organisations. Six trained bachelor’s and master’s psychology students supported data collection, which was carried out using the snowball sampling method. The procedure and the inclusion and ethics criteria for recruitment were the same as those for Study 1. We collected 600 questionnaires, which were then carefully examined. Only those filled out by individuals aged between 25 and 66 years and without missing data on the scales were included in this study.^2^

The final sample comprised 484 participants: 239 men (49.4%), 242 women (50%) and three people (0.6%) who did not indicate their gender. The mean age of the participants was 43.1 years (*SD* = 10). In total, 36 individuals (7.4%) had completed compulsory education, 277 (57.2%) had an upper-secondary diploma, and 168 (34.7%) had a college degree (three missing data). Further, 273 respondents (56.4%) were employed as clerical workers, 56 (11.6%) were blue-collar workers, 20 (4.1%) were health professionals, 40 (8.3%) were schoolteachers, 48 were employed in the service sector (9.9%), and 38 individuals (7.9%) held a middle-management position (nine missing values). The average tenure was equal to 19.5 years (*SD* = 10.9). Most participants (*n* = 350, 72.3%) had a permanent full-time job position, 71 (14.7%) had a permanent part-time job position, and 56 (11.6%) had a fixed-term employment contract (seven missing values). The majority (*n* = 311, 64.3%) reported having children (four missing values), and 348 (71.9) were married or cohabiting and mostly lived in Northeast Italy (*n* = 407, 84.1%). Again, data collection was completed before the sanitary emergency caused by SARS-COV-2 (COVID 19).

### Measures

#### Work-Home Culture Scale

We used the 18-item tool as in Study 7.

#### Work-Family Conflict Scale

This measures how often an individual’s participation in family roles is made more difficult by his/her participation in work roles. It is made up of five items (e.g., “Due to work-related duties, I have to make changes to my plans for family activities”), with a seven-point Likert-type scale that ranged from 1 (completely disagree) to 7 (completely agree) (Netemeyer et al., 1996) (Italian version by Colombo & Ghislieri, 2008).

#### Work-Family Enrichment Scale

This assesses the extent to which experiences in the work domain improve the quality of life in the family domain. It comprises three items (e.g., “At work, I feel positive emotions, and this helps me be a better family member”), with a seven-point Likert-type response scale that ranges from 1 (completely disagree) to 7 (completely agree) (Carlson et al., 2006) (Italian short version by Ghislieri et al., 2011).

#### Work-Family Balance Scale

This refers to the extent to which an individual is able to meet the negotiated role-related expectations in both the work and the family domain. It comprises six items (e.g., “I am able to negotiate and accomplish what is expected of me at work and in my family”), with a seven-point Likert-type response scale that ranges from 1 (completely disagree) to 7 (completely agree) (Carlson et al., 2009) (Italian version by Landolfi & Lo Presti, 2020).

#### Life Satisfaction Scale

We used the measure developed by Diener et al. (1985). One example of its five items is “In most ways, my life is close to my ideal”. The response scale ranged from 1 (completely disagree) to 7 (completely agree) (Italian version by Di Fabio & Gori, 2016).

#### Job Satisfaction Scale

We employed the Italian six-item scale proposed by Dazzi et al. (1998). An item example is “I feel satisfied with my job”. The response scale ranged from 1 (completely disagree) to 7 (completely agree).

#### Scale of Positive and Negative Experience (SPANE)

This measure, developed by Diener et al. (2010), is a 12-item scale, with six items devoted to positive experiences (e.g., “good”) and six items designed to assess negative experiences (e.g., “sad”). The participants were asked to evaluate how often they experienced each feeling on a scale ranging from 1 (very rarely or never) to 5 (very often or always). The positive and negative facets were scored separately. The summed positive and negative scores (SPANE-P and SPANE-N) can range between 6 and 30. The two scores can also be combined by subtracting the negative score from the positive one, and the resulting SPANE-B score can range between -24 and 24. A positive score indicates positive affective experiences, while a negative score indicates negative affective experiences.

#### Socio-Demographics Variables

These were assessed through the self-reporting of gender, age, geographic area of residence, education, employment status and tenure.

### Data Analysis

Two CFA models (the five-factor model and the two-second-order-factor model; see M2a and M4a in Table 1) were tested in order to check the construct validity of the WHC scale in a different sample using the same statistical package, analytic procedure and fit indices as those used in Study 7. The CFA was also used to estimate the goodness-of-fit of a three-factor model, which was made up of work-family conflict, work-family enrichment and work-family balance. SPSS 27 was used for descriptive analyses, reliability estimates through Cronbach’s alpha, Pearson’s correlation coefficients and hierarchical regression analyses. The hypothesised mediation models were tested via the PROCESS procedure (Hayes, 2013), adopting bias-corrected bootstrap 95% confidence intervals (with 1000 resampling), to determine the significance of the indirect effects of the five dimensions of WHC on the three components of subjective well-being (i.e., life and job satisfaction and positive affect) through the aspects of the work-family interface (i.e., conflict, enrichment and balance).

## Results

### Preliminary Analyses

The CFAs indicated that most of the fit indices of the five-factor model and the two-second-order-factor model were acceptable, similar to Study 1 (five-factor model: χ^2^_125_ = 749.96, *p* ≅ 00, RMSEA = 0.10, 90% CI: [0.09, 0.11], CFI = 0.95, SRMR = 0.06, AIC = 841.96; two second-order and five first-order factors model: χ^2^_129_ = 773.78, *p* ≅ 00, RMSEA = 0.10, 90% CI: [0.09, 0.11], CFI = 0.94, SRMR = 0.07, AIC = 857.78). The only fit index that was not satisfactory in both models was RMSEA, mirroring the evidence from Study 1. Freeing the covariance between the errors of the same three pairs of items (i.e., 1–2, 5–6, 12–13), which emerged as problematic also in Study 7, determined a goodness-of-fit improvement: five-factor model: χ^2^_122_ = 507.36, *p* ≅ 00, RMSEA = 0.08, 90% CI: [0.07, 0.09], CFI = 0.96, SRMR = 0.05, AIC = 605.36; two second-order and five first-order factors model: χ^2^_126_ = 542.70, *p* ≅ 00, RMSEA = 0.08, 90% CI: [0.07, 0.09], CFI = 0.96, SRMR = 0.06, AIC = 632.70. As in Study 7, the model with the lowest AIC was the one with five correlated factors.

Regarding the work-family interface, a measurement model with three latent factors (i.e., conflict, enrichment and balance) and 14 observed variables was estimated, obtaining satisfactory goodness-of-fit indices: χ^2^_74_ = 325.72, *p* ≅ 00, RMSEA = 0.08, 90% CI: [0.075, 0.093], CFI = 0.96, SRMR = 0.057, AIC = 387.72. All items showed significant loadings (between 0.65 and 0.85) on their expected factor. Work-family conflict was found to be negatively correlated with work-family enrichment (*ϕ* = -0.18, *p* < 0.001) and work-family balance (*ϕ* = -0.25, *p* < 0.001); work-family enrichment positively correlated with work-family balance (*ϕ* = 0.49, *p* < 0.001). In any case, the 95% confidence intervals, obtained by considering two standard errors above and below the coefficients, did not include the perfect correlation (i.e., 1.00), thus supporting the fact that all the measures captured distinct constructs (Bagozzi, 1994).

### Regression analyses

Table 3 presents descriptive statistics, Cronbach’s and Pearson’s correlations coefficients for the five first-order dimensions of the WHC scale, the three constructs of the work–family interface, and the outcome variables. All reliability estimates were satisfactory.

**Table 3:** Descriptive statistics, reliability estimates and correlations (*n* = 484)

Variables	*M* (*SD*)	1	2	3	4	5	6	7	8	9	10	11
1.Organisation’s support	4.29 (1.42)	0.92										
2.Colleagues’ support	4.56 (1.27)	0.47**	0.88									
3.Supervisor’s support	3.90 (1.43)	0.67**	0.52**	0.86								
4.Organisational time demands	3.99 (1.50)	-0.28**	-0.02	-0.13**	0.84							
5.Career consequences	4.14 (1.31)	-0.26**	-0.07	-0.20**	0.60**	0.82						
6.Work-family conflict	3.34 (1.33)	-0.30**	-0.14**	-0.22**	0.39**	0.29**	0.87					
7.Work-family enrichment	4.23 (1.41)	0.50**	0.28**	0.45**	-0.09	-0.11*	-0.15**	0.84				
8.Work-family balance	5.11 (0.90)	0.32**	0.32**	0.34**	0.07	0.04	-0.23**	0.43**	0.89			
9.Life satisfaction	4.23 (1.17)	0.24**	0.23**	0.27**	0.01	0.01	-0.12*	0.37**	0.40**	0.84		
10.Job satisfaction	4.46 (1.25)	0.42**	0.26**	0.39**	-0.25**	-0.18**	-0.30**	0.59**	0.35**	0.45**	0.86	
11. Positive affect (SPANE-B)	6.95 (7.03)	0.27**	0.18**	0.25**	-0.07	-0.05	-0.23**	0.29**	0.37**	0.51**	0.46**	0.88

Alpha coefficients on the main diagonal. Scores ranged from 1–7 for all variables but SPANE-B, which ranged from -24 to 24. * *p* < 0.05, ** *p* < 0.01.

To test the hypotheses H5 to H12, we conducted a series of multiple regression analyses. No multicollinearity issues emerged. The first three analyses used the five dimensions of the WHC as predictors and work-family conflict, work-family enrichment and work-family balance as dependent variables. Socio-demographics were added as control variables: age, gender (0 = men, 1 = women) and parental status (0 = no children, 1 = children). The results showed that *organisation’s support* was negatively associated with work-family conflict (*b* = -0.13, *p* < 0.03, 95% CI: [-0.25, -0.01]) and positively associated with work-family enrichment (*b* = 0.35, *p* < 0.0001, 95% CI: [0.20, 0.48]) and work-family balance (*b* = 0.11, *p* < 0.005, 95% CI: [0.03, 0.19]). *Colleagues’ support* was positively associated only with work-family balance (*b* = 0.10, *p* < 0.006, 95% CI: [0.02, 0.18]). *Supervisor’s support* was positively associated with work–family enrichment (*b* = 0.22, *p* < 0.0001, 95% CI: [0.09, 0.35]) and work–family balance (*b* = 0.10, *p* < 0.007, 95% CI: [0.02. 0.18])*. Organisation’s time demands* was positively associated only with work–family conflict (*b* = 0.28, *p* < 0.0001, 95% CI: [0.18, 0.37]). Finally, *career consequences* did not present any significant relationship with the three dependent variables. None of the socio-demographic variables were associated with dependent variables. The five dimensions of the WHC altogether explained 20% of the work–family conflict variance (*F*_8,466_ = 14.23, *p* < 0.0001), 27% of the work–family enrichment variance (*F*_8,466_ = 21.67, *p* < 0.0001) and 18% of work–family balance (*F*_8,466_ = 12.65, *p* < 0.0001).

Next, we conducted three hierarchical regression analyses with life satisfaction, job satisfaction and positive affect (SPANE-B) as the outcome variables, respectively. The control variables and the five dimensions of the WHC were entered in the first step. In the second step, the hypothesised mediators (work-family conflict, work-family enrichment and work-family balance) were considered. Regarding life satisfaction, the hypothesised regression model explained 24% of its variance (*F*_11,463_ = 12.91, *p* < 0.0001). Among the socio-demographic variables, only parental status presented a positive relationship in all steps (Step 1, *b* = 0.44, *p* < 0.002, 95% CI: [0.18, 0.69]; Step 2, *b* = 0.37, *p* < 0.005, 95% CI: [0.12, 0.60]): participants with children reported being more satisfied with their life. In Step 1, only *supervisor’s support* showed a significant total effect on life satisfaction. In Step 2, work-family enrichment (*b* = 0.18, *p* < 0.0001, 95% CI: [0.09, 0.27]) and work-family balance (*b* = 0.35, *p* < 0.0001, 95% CI: [0.22, 0.46]) were significantly associated with life satisfaction. The parallel mediation analysis, which considered all the supposed mediators, indicated that the direct effect of *supervisor’s support* was not significant, while two specific indirect effects were significant: the first one was totally mediated by work-family enrichment, and the second by work-family balance (Table 4).

**Table 4:** Mediation analysis: Total, direct and specific indirect effects

Model	Total effects	Direct effects	Specific indirect effects	Mediation
SS → WFE → Life satisfaction	0.12* 95% CI: [0.01, 0.23]	0.05 ns	0.04** 95% CI: [0.01, 0.07]	total
SS → WFB → Life satisfaction	0.12* 95% CI: [0.01, 0.23]	0.05 ns	0.04* 95% CI: [0.01, 0.07]	total
OS → WFE → Job satisfaction	0.19*** 95% CI: [0.07, 0.31]	0.02 ns	0.14*** 95% CI: [0.10, 0.20]	total
SS → WFE → Job satisfaction	0.17*** 95% CI: [0.06, 0.28]	0.07 ns	0.09* 95% CI: [0.04, 0.15]	total
Time → WFC → Job satisfaction	-0.12** 95% CI: [-0.22, -0.03]	-0.11** 95% CI: [-0.20, -0.03]	-0.03** 95% CI: [-0.06, -0.01]	partial
OS → WFB → SPANE-B	0.86** 95% CI: [0.22, 1.57]	0.38 ns	0.21* 95% CI: [0.05, 0.41]	total

OS = *organisation’s support*, SS = *supervisor’s support*, Time = *organisational time demands*, WFC = work–family conflict, WFE = work–family enrichment, WFB = work–family balance, SPANE-B = positive affect*.* Unstandardised coefficients are reported. *p < 0.05, **p < 0.01, ***p < 0.001.

The same regression model explained 43% of the variance of job satisfaction (*F*_11,463_ = 31.34, *p* < 0.0001). In Step 1, *organisation’s support*, *supervisor’s support* and *organisational time demands* showed significant totals effects on job satisfaction (Table 4). In Step 2, work-family conflict (*b* = -0.12, *p* < 0.002, 95% CI: [-0.21, -0.05]), work-family enrichment (*b* = 0.40, *p* < 0.0001, 95% CI: [0.32, 0.49]) and work-family balance (*b* = 0.13, *p* < 0.04, 95% CI: [0.01, 0.25]) were significantly associated with job satisfaction. The parallel mediation analysis demonstrated that the direct effects of *organisation’s support* and *supervisor’s support* were not significant (Table 4). Work-family enrichment totally mediated the relationships between *organisation’s support* and job satisfaction and between *supervisor’s support* and the same outcome variable. *Organisational time demands* showed a significant direct effect on job satisfaction, but the indirect effect of *organisational time demands* through work-family conflict was also found to be significant (Table 4). Thus, work-family conflict partially mediated the relationship between *organisational time demands* and job satisfaction.

Regarding the positive affect, as measured by SPANE-B scores, the hypothesised regression model explained 19% of variance of this outcome variable (*F*_11,463_ = 9.79, *p* < 0.0001). Gender presented a significant relationship in all steps (Step 1, *b* = -1.56, *p* < 0.02, 95% CI: [-2.81, -0.35]; Step 2, *b* = -1.52, *p* < 0.02, 95% CI: [-2.76, -0.32]) with this outcome variable: women reported experiencing a less positive affect than men did. Only *organisation’s support* presented a significant total effect on positive affect. In Step 2, work-family conflict (*b* = -0.72, *p* < 0.005, 95% CI: [-1.33, -0.06]) and work-family balance (*b* = 1.89, *p* < 0.0001, 95% CI: [0.92, 2.80]) were significantly associated with positive affect. The parallel mediation analysis showed only one significant specific indirect effect: work-family balance totally mediated the relationship between *organisation’s support* and positive affect (Table 4).

## Conclusion

The results of the CFAs reinforced the factorial structure of the WHC scale that had already emerged in Study 1, and the three-factor model made up of work–family conflict, enrichment and balance. In this last case, the distinctiveness of the three factors received support, and their correlations were in line with the extant literature (Carlson et al., 2009; Landolfi & Lo Presti, 2020).

Our hypotheses regarding the relationships between the five dimensions of the WHC and the three aspects of the work-family interface (H5-H12) were partially supported. Among the three dimensions of work-home support, only *organisation’s support* was negatively associated with work-family conflict (H5). The same dimension was positively associated with work-family enrichment and work-family balance (H7 and H9). *Colleagues’ support* was positively associated only with work-family enrichment (H7), and *supervisor’s support* was positively associated with work-family enrichment and work-family balance (H7 and H9). Among the two dimensions of a hindering WHC, only that of *organisational time demands* was positively associated with work-family conflict (H6). The negative *career consequences* dimension was not associated with work-family interface when the other dimensions of the WHC were controlled. Work-family conflict, controlling for the other aspects of the work-home interface, was found to be negatively associated with job satisfaction and positive affect, which partially supports H11. As expected (H12), work-family enrichment and work-family balance were positively associated with all three components of subjective well-being.

The mediation analyses showed that work-family enrichment mediated the relationships between *supervisor’s support* and life and job satisfaction. It also mediated the relationship between *organisation’s support* and job satisfaction. Work-family balance mediated the relationship between *supervisor’s support* and life satisfaction and that between *organisation’s support* and positive affect. Finally, work-family conflict partially mediated the relationship between *organisational time demands* and job satisfaction (H13).

## General Discussion

The first aim of this paper was to offer a contribution to the adaptation and validation of the WHC scale by Dikkers et al. (2007) in the Italian context to enable its use in research projects that aim to compare data between organisations, nations and cultures and for interventions in the fields of work and organisational psychology and occupational health (Study 1). As far as we know, this is the first attempt to validate the whole Dikkers et al.’s (2007) scale in a different sociocultural context. To fulfil this objective, we conducted CFAs to check the adequacy of competing models of the latent structure of this scale. Despite both the five-first-order-factor and the two-second-order-factor models presented quite a similar fit to the data, the solution with five correlated factors was retained. The reliability of the five first-order factors and two second-order factors were all satisfactory. Therefore, researchers and practitioners may choose to compute five instead of two composite scores depending on the rationale of their study or organisational intervention. The five first-order factors structure turned out to be mostly invariant when comparing different subgroups of workers (based on gender, parental status and employment in a public *vs* private organisation). Therefore, we consider our hypotheses H1 and H2 to be largely supported.

Workers from public organisations perceived the WHC of their organisations as being less supportive and less obstructive to the establishment of a balance between work and home duties as compared with employees of private organisations, which partially supports hypothesis H3. In other words, employees of public organisations reported less *time demands* and less negative *career consequences* related to their efforts to manage both work and family duties. However, at same time, they felt less supported by both their organisations and their supervisors. This finding suggests that organisations that are owned or controlled by the government – in which employment contracts and careers are often statutory-regulated – seem to be less demanding towards employees (e.g., asking them to work overtime on a regular basis, to be available all of the time or to put their job before their private life). At the same time, employees of public organisations believed that the use of family-friendly benefits would not jeopardise their professional future. Moreover, organisations and supervisors in the public sector are perceived to be less supportive and sensitive towards the integration of employees’ work and family duties and the utilisation of work-family arrangements compared to private organisations. Even if the law grants certain work-family arrangements, it is likely that employees belonging to the private sector will need to negotiate their access to family-friendly benefits with their supervisors more than those belonging to the public sector do. Thus, the role and receptivity of supervisors can be considered more crucial in the private sector *vs* public sector.

Women reported experiencing less *organisational time demands* and negative *career consequences* for using family-friendly benefits as compared to men, and this does not support H4. In line with the other findings in the literature (e.g., Beauregard, 2010; Dikkers et al., 2004), this can be explained by referring to the gender role theory, which states that social pressures encourage people to behave in ways that are consistent with their prescribed gender roles (Eagly, 1987). Given that men are subject to the gender role expectation of fulfilling the “breadwinner” role, they could have experienced work demands as being more dominant than home demands and suffered stronger penalties than women for not complying with work role expectations and for trying to reconcile work and family responsibilities.

The purpose of Study 2 was to investigate how the WHC was associated with employees’ subjective well-being, and whether the work-family interface represents a mechanism, through which the organisational culture exerts an impact on the same. Our findings showed that the experience of a supportive WHC, with regard to work-family issues and the use of family-friendly benefits, was significantly related to the work-family interface. As expected, employees who experienced support from their organisations expressed less work-family conflict, which is in line with H5, and a higher level of work-family enrichment and balance, which is in line with H7 and H9. In addition, employees who received support from their supervisor experienced higher work–family enrichment and balance, thus further supporting H7 and H9. Finally, employees who received support from their colleagues perceived higher work-family balance, which is, again, in favour of H9. In sum, supportive organisations and supervisors are the most important resources that can facilitate the experience of a positive work-family interface. Support from colleagues in using family-friendly benefits does not seem to be beneficial in mitigating work-family conflict or in increasing work-family enrichment; however, it seems to help employees effectively fulfil both work and family duties. Only a high level of *organisational time demands* was associated with a high level of work-family conflict, partially supporting H6. H8 and H10 were not supported by data. These findings are coherent with the extant literature which examined these relations using the work-family scale by Thompson et al. (1999) or some subscales of the WHC scale (e.g., Baral & Bhargava, 2010; Beham & Drobnič, 2010; Beham et al., 2011, 2014; Dikkers et al., 2007).

As hypothesised, and in agreement with the literature, almost all the associations between the three aspects of the work-family interface and subjective well-being were found to be significant and in the expected direction (see H11 and H12). Lastly, in line with the studies of Beauregard (2011) and Mauno et al. (2005), our findings indicated that the WHC exhibits significant associations with subjective well-being and that these associations are largely indirect rather than direct (H13). In other words: (a) supervisor’s support enhanced the perceived work-family enrichment of employees, which, in turn, increased life and job satisfaction; (b) the same dimension increased employees’ perception of work-family balance, which, in turn, increased life satisfaction; (c) organisation’s support was positively associated with work-family enrichment and balance, which, in turn, enhanced both job satisfaction and positive affect. Thus, work-family enrichment and work-family balance are the aspects of the work-family interface that appear to be more involved in the mechanism through which organisations’ and supervisors’ support can exert a positive impact on an employee’s subjective well-being. The mediation analysis indicated only one significant direct effect linking *organisational time demands* with job satisfaction. Consistent with the resources-demands perspective, organisations’ expectation that employees should prioritise their work over their family and spend a considerable amount of time visibly at work resulted in decreased employee job satisfaction. At the same time, the continuous experience of workload increased work-family conflict, leading, again, to a decrease in job satisfaction.

In conclusion, organisations and supervisors who offer support to their employees with respect to using family-friendly arrangements seem to be able to improve subordinates’ subjective well-being, which arises from establishing a positive relationship (marked by perceptions of both enrichment and balance) between work and family duties. From an application point of view, organisations that aim to increase workers’ well-being should also consider the employees’ experience of the work-family interface. Moreover, they should create a family-supportive environment that encourages employees to use the work-family arrangements that are available in the organisation. Although, in practice, such a change in culture may be difficult to achieve, training managers and supervisors regarding the implementation of family-friendly policies and improving their awareness of the associated benefits can be worthwhile, as it may actively contribute to the retention of a more satisfied workforce.

## Limitations

Certain limitations of our studies should be noted. First, the two studies relied exclusively on self-reported measures, which might have led to an overestimation of the associations between the variables due to common method variance. However, the guarantee of anonymity and the reduction of people’s evaluation apprehension by assuring them that there are no right or wrong answers may have attenuated this issue (Podsakoff et al., 2003). Second, self-reported measures can be affected by social desirability bias even though granting anonymity during data collection usually helps counterbalance this weakness. Third, as highlighted by Brough et al. (2014), another limitation is associated with the fact that the items that measured the work-family interface place an emphasis on family, excluding other potential non-family and non-work commitments. For instance, the emphasis on family makes it problematic to use the measures of balance for people who have no immediate family members (single people and/or those who do not have children) but do need to achieve an appropriate balance between their work and life duties. Fourth, the use of a cross-sectional design does not allow us to make any causal inference about the relations between the WHC and the other variables. Finally, we interviewed convenience samples of workers, and, although the samples were quite large, our results cannot be generalised to the entire Italian workers population.

## Appendix 1

Table A

**Table A:** Study 1: WHC scale, five-correlated-factor model and standardised factor loadings (*n* = 784)

Items	Organisation’s support	Colleagues’ support	Supervisor’s support	Time demands	Career consequences
1. Managers in this organization are generally considerate towards the private life of employees. [*Nella mia azienda/organizzazione, in genere, i dirigenti mostrano rispetto per la vita privata dei dipendenti*]	0.89				
2. In this organization, people are sympathetic towards care responsibilities of employees. [*Nella mia azienda/organizzazione, le persone mostrano comprensione per le responsabilità di cura che i dipendenti hanno nella propria vita privata*]	0.90				
3. In this organization it is considered important that, beyond their work, employees have sufficient time left for their private life. [*Nella mia azienda/organizzazione si ritiene importante che, oltre che per il lavoro, i dipendenti abbiano tempo sufficiente per la propria vita privata*]	0.81				
4. This organization is supportive of employees who want to switch to less demanding jobs for private reasons. [*La mia azienda/organizzazione sostiene i dipendenti che vogliono passare a lavori o mansioni meno impegnativi per motivi privati*]	0.66				
5. I am comfortable in discussing aspects of my private life with my colleagues. [*Mi sento a mio agio a parlare di aspetti della mia vita privata con i miei colleghi*]		0.63			
6. My colleagues help me out when I am (temporarily) preoccupied with my care responsibilities. [*I miei colleghi mi aiutano quando sono (temporaneamente) preoccupato/a per le mie responsabilità di cura nella vita privata*]		0.71			
7. My colleagues support employees who (temporarily) want to reduce their working hours for private reasons. [*I miei colleghi sostengono i dipendenti che vogliono passare a lavori o mansioni meno impegnativi per motivi privati*]		0.87			
8. My colleagues support employees who (temporarily) want to reduce their working hours for private reasons. [*I miei colleghi sostengono i dipendenti che (temporaneamente) vogliono ridurre le loro ore lavorative per motivi privati*]		0.85			
9. I am comfortable in discussing my private life with my superior. [*Mi sento a mio agio nel parlare della mia vita privata con il mio superiore*]			0.61		
10. My superior supports employees who (temporarily) want to reduce their working hours for private reasons. [*Il mio superiore sostiene i dipendenti che (temporaneamente) vogliono ridurre le loro ore di lavoro per motivi privati*]			0.93		
11. My superior supports employees who want to switch to less demanding jobs for private reasons. [*Il mio superiore sostiene i dipendenti che vogliono passare a lavori meno impegnativi per motivi privati*]			0.91		
12. To get ahead at this organization, employees are expected to work overtime on a regular basis. [*Nella mia azienda/organizzazione ci si aspetta che, per andare avanti, i dipendenti svolgano regolarmente lavoro straordinario*]				0.66	
13. In order to be taken seriously in this organization, employees should work long days and be available all of the time. [*Al fine di essere presi sul serio nella mia azienda/organizzazione, i dipendenti dovrebbero lavorare molte ore al giorno ed essere disponibili per tutto il tempo*]				0.90	
14. In this organization, employees are expected to put their job before their private life when necessary. [*Nella mia azienda/organizzazione ci si aspetta che i dipendenti, se necessario, mettano il loro lavoro prima della loro vita privata*]				0.82	
15. Employees who (temporarily) reduce their working hours for private reasons are considered less ambitious in this organization. [*Nella mia azienda/organizzazione i dipendenti che (temporaneamente) riducono le ore di lavoro per motivi privati sono considerati meno ambiziosi*]					0.80
16. To turn down a promotion for private reasons will harm one’s career progress in this organization. [*Nella mia azienda/organizzazione, rifiutare una promozione per motivi privati danneggia il proprio sviluppo di carriera*]					0.78
17. Employees who (temporarily) reduce their working hours for private reasons are less likely to advance their career in this organization. [*Nella mia azienda/organizzazione, i dipendenti che (temporaneamente) riducono le ore lavorative per motivi privati hanno meno probabilità di fare carriera*]					0.79
18. In this organization, it is more acceptable for women to (temporarily) reduce their working hours for private reasons than for men. [*Nella mia azienda/organizzazione, è più accettabile che le donne riducano (temporaneamente) le ore di lavoro per motivi privati piuttosto che gli uomini*]					0.41

## Appendix 2

Table B

**Table B:** Study 1: WHC scale, five-correlated-factor model. Results of multi-group analyses

Multigroup comparisons	Fit indices	Test of invariance
*Gender*		
Configural invariance	χ^2^(250) = 1246.34, *p* = 0.00 CFI = 0.94, RMSEA = 0.10, SRMR = 0.06	
Metric invariance	χ^2^(263) = 1263.77, *p* =0.00 CFI = 0.94, RMSEA = 0.09, SRMR = 0.06	M1–M0 Δχ^2^(13) = 17.43, *ns* ΔCFI = 0.00, ΔRMSEA = -0.001 ΔSRMR = -0.002
Factor covariance invariance	χ^2^(278) = 1278.31, *p* = 0.00 CFI = 0.94, RMSEA = 0.09, SRMR = 0.07	M2–M1 Δχ^2^(15) = 14.54, *ns* ΔCFI = 0.00, ΔRMSEA = -0.003 ΔSRMR = 0.005
Residual invariance	χ^2^(296) = 1292.31, *p* = 0.00 CFI = 0.94, RMSEA = 0.09, SRMR = 0.07	M3–M2 Δχ^2^(18) = 14, *ns* ΔCFI = 0.00, ΔRMSEA = -0.003 ΔSRMR = 0.000
*Parental status*		
Configural invariance	χ^2^(250) = 1290.41, *p* = 0.00 CFI = 0.94, RMSEA = 0.10 SRMR = 0.07	
Metric invariance	χ^2^(263) = 1302.13, *p* = 0.00 CFI = 0.94, RMSEA = 0.10, SRMR = 0.07	M1–M0 Δχ^2^(13) = 11.72 ns ΔCFI = 0.00, ΔRMSEA = 0.000 ΔSRMR = -0.002
Factor covariance invariance	χ^2^(278) = 1328.92, *p* = 0.00 CFI = 0.94, RMSEA = 0.09, SRMR = 0.08	M2–M1 Δχ^2^(15) = 26.79, *p* = 0.03 ΔCFI = 0.00, ΔRMSEA = -0.002 ΔSRMR = 0.004
Residual invariance	χ^2^(296) = 1350.60, *p* = 0.00 CFI = 0.94, RMSEA = 0.09, SRMR = 0.07	M3–M2 Δχ^2^(18) = 21.68, *ns* ΔCFI = 0.00, ΔRMSEA = -0.002 ΔSRMR = -0.001
*Public-sector vs private-sector workers*		
Configural invariance	χ^2^(250) = 1216.00 *p* = 0.00 CFI = 0.94, RMSEA = 0.10, SRMR = 0.06	
Metric invariance	χ^2^(263) = 1225.15, *p* = 0.00 CFI = 0.94, RMSEA = 0.10, SRMR = 0.07	M1–M0 Δχ^2^(13) = 9.15, *ns* ΔCFI = 0.00, ΔRMSEA = 0.000 ΔSRMR = 0.002
Factor covariance invariance	χ^2^(278) = 1268.66, *p* = 0.00 CFI = 0.93, RMSEA = 0.09, SRMR = 0.08	M2–M1 Δχ^2^(15) = 43.51, *p* = 0.0002 ΔCFI = -0.01, ΔRMSEA = -0.001 ΔSRMR = 0.015
Residual invariance	χ^2^(296) = 1336.19, *p* = 0.00 CFI = 0.93, RMSEA = 0.09, SRMR = 0.08	M3–M2 Δχ^2^(18) = 67.53, *p* = 0.0000 ΔCFI = 0.00, ΔRMSEA = 0.00 ΔSRMR = 0.00

## Footnotes

1 All questionnaires were scrutinized in order to assess the correspondence with the inclusion criteria (e.g., age range, presence of both colleagues and a supervisor, job type and employment contract), using also some control questions that were not considered in 12. We have also eliminated a limited number of outliers.

2 The same screening procedure as concerns inclusion criteria and outliers used for Study 1 was followed.
